# Target enhanced 2D similarity search by using explicit biological activity annotations and profiles

**DOI:** 10.1186/s13321-015-0103-5

**Published:** 2015-11-17

**Authors:** Xiang Yu, Lewis Y. Geer, Lianyi Han, Stephen H. Bryant

**Affiliations:** National Center for Biotechnology Information, National Library of Medicine, National Institutes of Health, 8600 Rockville Pike, Bethesda, MD 20894 USA

**Keywords:** 2D similarity search, Iterative similarity search, Nearest neighbor, Iterative similarity search with classification, Profile

## Abstract

**Background:**

The enriched biological activity information of compounds in large and freely-accessible chemical databases like the PubChem Bioassay Database has become a powerful research resource for the scientific research community. Currently, 2D fingerprint based conventional similarity search (CSS) is the most common widely used approach for database screening, but it does not typically incorporate the relative importance of fingerprint bits to biological activity.

**Results:**

In this study, a large-scale similarity search investigation has been carried out on 208 well-defined compound activity classes extracted from PubChem Bioassay Database. An analysis was performed to compare the search performance of three types of 2D similarity search approaches: 2D fingerprint based conventional similarity search approach (CSS), iterative similarity search approach with multiple active compounds as references (ISS), and fingerprint based iterative similarity search with classification (ISC), which can be regarded as the combination of iterative similarity search with active references and a reversed iterative similarity search with inactive references. Compared to the search results returned by CSS, ISS improves recall but not precision. Although ISC causes the false rejection of active hits, it improves the precision with statistical significance, and outperforms both ISS and CSS. In a second part of this study, we introduce the profile concept into the three types of searches. We find that the profile based non-iterative search can significantly improve the search performance by increasing the recall rate. We also find that profile based ISS (PBISS) and profile based ISC (PBISC) significantly decreases ISS search time without sacrificing search performance.

**Conclusions:**

On the basis of our large-scale investigation directed against a wide spectrum of pharmaceutical targets, we conclude that ISC and ISS searches perform better than 2D fingerprint similarity searching and that profile based versions of these algorithms do nearly as well in less time. We also suggest that the profile version of the iterative similarity searches are both better performing and potentially quicker than the standard algorithm.

**Electronic supplementary material:**

The online version of this article (doi:10.1186/s13321-015-0103-5) contains supplementary material, which is available to authorized users.

## Background

Large scale virtual screening methods have been an attractive approach for prescreening millions of compounds in commercial or public chemical databases to find compounds specifically active against a specific target, especially in early stages of modern drug development pipelines. Among the search methods available, 2D fingerprint based conventional similarity search (CSS) is a well-established virtual screening tool [1, 2], in which the similarities between database compounds and the query compound are measured and ranked, and hits are selected from the top of the ranked list. The central principle underlying virtual screening methods is the molecular similarity principle, which states that structurally similar small molecules tend to express similar biological activities [2–4]. A molecular 2D fingerprint is usually defined as a fixed-length bit string where each bit represents a specific molecular substructure feature or structure property. As a ligand based virtual screening method, the generation of molecular 2D fingerprint only requires the molecular graph as input. The similarity between the input and compound being searched is usually measured by the Tanimoto coefficient [5], one of the most common approaches for database searching due to its simplicity [6–8], fast speed, easy implementation and results in drug discovery [8–10].

Despite the development of more sophisticated 3D similarity approaches [11, 12] and machine learning methods such as random forests, naïve Bayesian classifiers, and support vector machines, 2D similarity search continues to be the focus of virtual screening research to better retrieve compounds of desired bioactivities or physical properties [13–17]. In part, this is due to the relative computational efficiency, which is important for large online chemical databases such as PubChem to answer user queries in a reasonable amount of time. These advanced 2D similarity search strategies generally can be summarized into three categories. The first category is data fusion of similarity coefficients, in which several types of similarity coefficients take into account different characteristics of compounds that are combined together to optimize the measure of compound similarity [16, 17]. The second category of search strategies is non-iterative single reference searches that are often that based on one-against-one similarity measures, i.e., bit-weighting [18, 19] and bit-truncation [20] approaches. The third category is the iterative similarity search with multiple references, which is also known as nearest neighbor (NN) search or turbo search [10, 14, 21–24]. ISS is an iterative similarity search approach in which the similarity of a database compound is determined by comparing the query compound to multiple references with the same biological activity. The basic theory behind ISS is that the neighbor list of references map out a hypervolume in the multidimensional sampling space for the bioactivity of interest, and consequently the top-ranked structures in the search result are more likely to be compounds with similar biological activity. Peter Willett et al. compared ISS with CSS and bit-weighting approaches, and they found an overwhelming advantage of ISS in retrieving active hits [10]. Furthermore, accumulative simulations have also demonstrated that ISS with the MAX fusing rule (maximum of all of similarity pairs) usually gets better search results than ISS with the SUM fusing rule [10, 22, 25]. Overall, by using multiple compounds as “baits” to fish out more active compounds against a given target from a database of decoys, this simple but efficient approach for target enhanced similarity search is promising for chemical database screening.

One of the objectives in 2D similarity searches is to improve the recall performance. This is based on a general assumption that if more active hits are included in the hit list, then the there is a higher probability that the remaining hits in the hit list may share the same biological activity. Nevertheless, constrained by the quality of the data [26], the number and nature of compounds in the data set [26], and more importantly the underlying limitation of molecular representations [27, 28], it is unavoidable to include inactive compounds in database screening based solely on the chemical similarity principle. Mounting evidence suggests that the previous assumption does not always work especially if “activity cliffs” widely exist in a given chemical space [29, 30]. Currently many chemical databases like PubChem Bioassay and ChEMBL preserve both active and inactive target-ligand information in each deposited assay [31]. Enriched active and inactive end-points enable us to not only re-evaluate the search performance of the ISS and the CSS by counting the numbers of annotated active and inactive hits in the hit lists, but also to utilize the structure information of these inactive compounds to reshape the chemical sampling space of the similarity search. If ISS has high specificity in retrieving active compounds, the reverse version of ISS by replacing active references in the neighbor list with inactive references should also retain the ability to identify inactive compounds. Ideally, the combination of ISS and the reversed ISS, which we call it as iterative search with classification or ISC in this study, may help to both retrieve active compounds and to purify the results from database screening.

The purpose of this study is to develop and compare target enhanced similarity search approaches. ChEMBL bioassay data [32] and PubChem confirmatory bioassay data [31] with explicit EC50, IC50 or Ki value were retrieved from PubChem Database, and the data was combined into 208 activity classes for our test. Each activity class corresponded to a protein target. In an effort to expand the sampling space and alleviate the computational burden of iterative searches, we also introduced the profile concept into target enhanced similarity search. In this case, the binary 2D fingerprints in the CSS, ISS and ISC were replaced by representative average profiles (AVEs). In total, 6 search approaches including 2 non-iterative approaches (2D fingerprint base d conventional similarity search or CSS, and average profile search or PBSS), 2 iterative ISS approaches with multiple active references (fingerprint based ISS, and average profile based ISS or PBISS search), and finally 2 iterative searches with classification (fingerprint based ISC, and average profile based ISC or PBISC) were systematically tested on 208 activity classes. The arithmetic mean of recall rates tested on the selected activity class (ARR), the arithmetic precision rate (APR), and area under the ROC curve (AUC) of each of 208 activity classes were compared to comprehensively evaluate the search performance of all 6 search approaches. The detailed data set preparation, description of search approaches and results of the search simulations are reported herein.

## Results and discussion

Our study attempts to address three questions: Can chemical similarity searches be improved by (1) using iterative searches, (2) classifying search results by using bioactivity data, and (3) by using fingerprint profiles? Furthermore, what is a reasonable metric for determining the answer to these questions—should we only measure recall, as has been typically done in other studies, or measure both recall and precision at the same time?

For these purposes, the recall, precision and comprehensive search performance (AUC) determined by calculating ARRs and APRs on 208 activity classes using 6 search approaches are compared and described below. The specific AUC, ARR and APR values of each activity class returned by six search approach can be found in three heatmaps in Additional file 1: Figure S4. It should be noted that since explicitly annotated inactives were added in each activity class, the precision rate calculation of each similarity search follows a new definition described in the method part below.

### Profiling of conventional similarity search on 208 activity classes

2D Fingerprint based similarity search has been very popular in various applications and it is often used as a standard search algorithm for benchmarking new algorithms. Therefore, we first characterized the search performance of the CSS search on 208 well-curated activity classes.

**Fig. 1:**
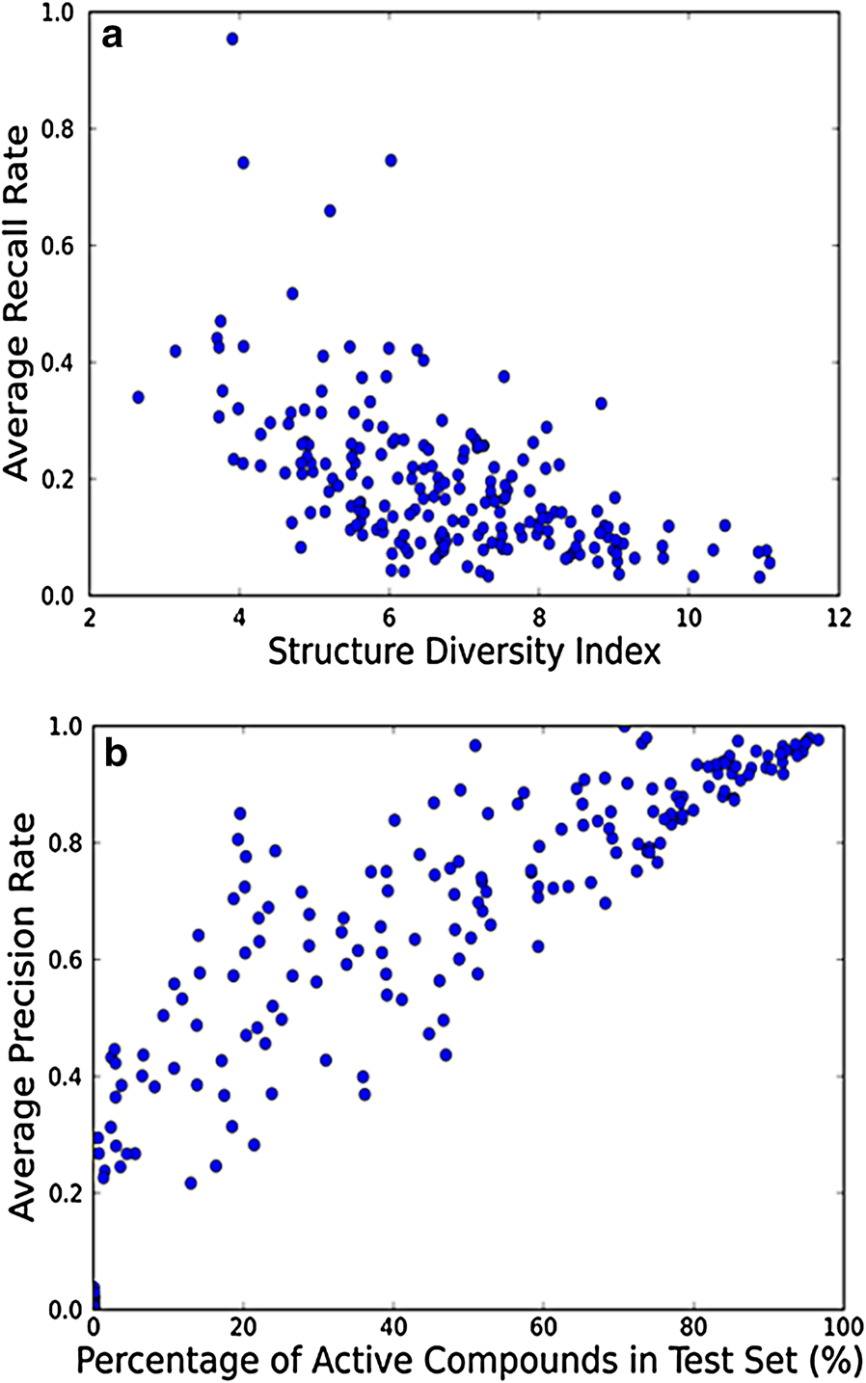
**a** Plot of average recall rates (ARRs) returned by CSS against I-index for 208 activity classes. **b** Plot of average precision rates (APRs) returned by CSS against the portion of active compounds in the test set

Figure 1a shows the ARRs of 208 activity classes against the structural diversity index of these activity classes, including 178 activity classes with their ARRs <0.3. Although the recall performance of a query is highly dependent on the enrichment of similar active compounds in the test set, it is likely that the higher structural diversity of active compounds of an activity class makes it more difficult to efficiently retrieve active hits when the number of hits is limited. Calculations of the average ARRs of CSS at different similarity cutoffs were carried out and the average ARR curve in Additional file 1: Figure S2 suggests that the CSS approach generally reached the maximal recall limitation in the top 1 % of hits. Although enrichment using similarity search (19.53 ± 14.2) is observed in our study (Table 1), CSS search searches on 178 of 208 activity classes return ARRs lower than 0.3, and only five activity classes (Class 45, 54, 61, 74, and 153) return ARRs greater than 0.5. This low recall rate means that the majority of hits in the hit list are compounds with undetermined bioactivity or with inactive bioactivity. On the other hand, the average precision rates (APRs) of 208 activity classes against the portion of actives in the test set is plotted in Fig. 1b, and most of points are above the diagonal of the figure, which confirms that the molecular similarity principle generally works in similarity search when retrieving compounds of similar bioactivity. However, the distribution of the points in Fig. 1b also indicates that the larger number of explicitly tested inactives in the test set, the higher probability of hitting an explicitly tested inactive compound. In the case of searching on activity classes such as class 19, 28, and 32, which each has more than 100,000 annotated inactive compounds in the test set, the APRs are all below 0.01. It means that even if the recall rate of the query is relatively acceptable, the inactive hits in the final hit list may overwhelm the active hits. In this situation, it is not likely that the compounds with uncertain bioactivity in the hit list share the desired bioactivity of the query compound. This result indicates that a high recall rate may not necessarily led to a high quality search similarity. We suggest that a good similarity search approaches should improve both recall and precision performance.

**Table 1:** Summary of average enrichments (AEFs), ARRs, APRs and AUCs of 208 activity classes

		CSS	PBSS	ISS	PBISS	ISC	PBISC
AEF	Mean	19.532	23.124	23.124	23.609	20.319	20.989
	Std	14.161	15.309	14.75	15.176	12.407	12.933
ARR	Mean	0.198	0.234	0.234	0.239	0.205	0.212
	Std	0.143	0.154	0.148	0.153	0.124	0.13
APR	Mean	0.594	0.591	0.593	0.593	0.626	0.625
	Std	0.321	0.324	0.323	0.322	0.333	0.33
AUC	Mean	0.568	0.638	0.666	0.666	0.703	0.708
	Std	0.101	0.115	0.119	0.118	0.12	0.118

### Compare iterative similarity search and iterative similarity search with classification to conventional similarity search

Because there is no obvious relationship between recall rate and precision rate observed in our analysis and a high portion of annotated inactive hits in the hit list are not our expected result, we regard recall and precision of equal importance in evaluating similarity search performance.

**Fig. 2:**
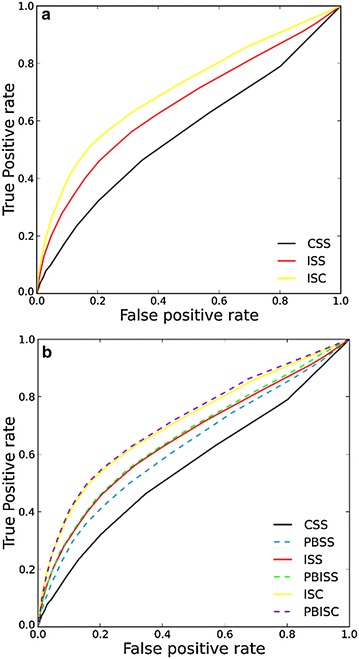
**a** ROC curves averaged from 208 activity classes returned by CSS, ISS, and ISC approaches. **b** Comparison of ROC curves of PBSS, PBISS, and PBISC to the corresponding Morgan fingerprint based CSS, ISS and ISC approaches

6 ROC plots averaged from area under receiver operating characteristic curves (AUCs) of 208 activity classes (Fig. 2) help us see the overall search performance under different false positive rates (FPRs). Solid lines in black, red and yellow colors are ROC plots for CSS, ISS and ISC respectively. ISC performs better than ISS and CSS in the whole graph whereas CSS approaches the diagonal of the ROC after FPR of 0.8. Although the ISC search approach uses about twice the computational resources of ISS on average, this approach does provides better search performance. On the other hand, ISC and ISS have limitations. For example, if there is no enriched bioactivity data available and active compounds belonging to the same activity classes are not structurally diverse, it is not possible to perform the ISC search and also we do not expect the search performance of ISC and ISS to be significantly better than CSS.

**Fig. 3:**
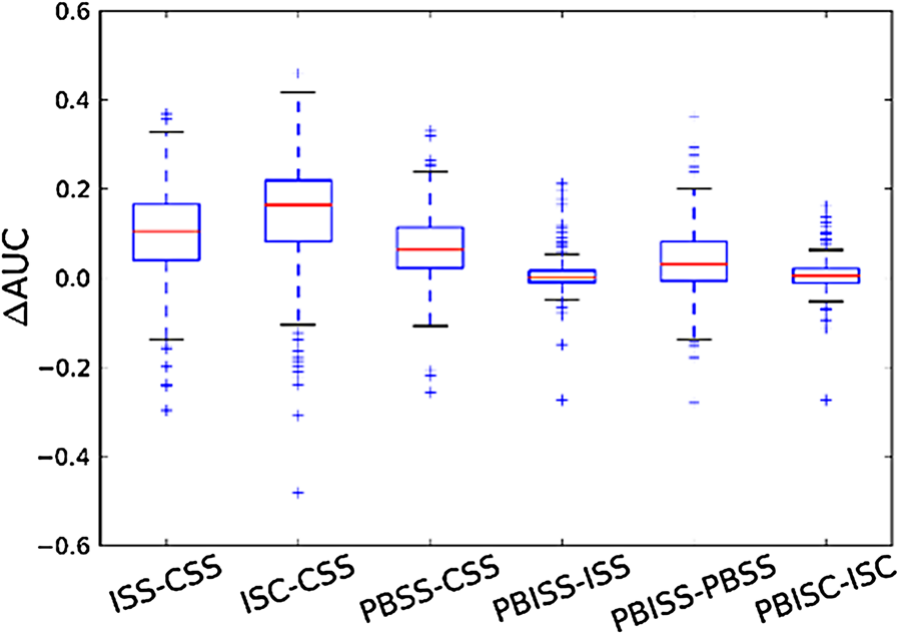
The boxplot of ∆AUC between ISS and CSS, ∆AUC between ISC and CSS, ∆AUC between PBSS and CSS, ∆AUC between PBISS and ISS, ∆AUC between PBISS and PBSS, and ∆AUC between PBISC and ISC

The AUCs of CSS, ISS and ISC approaches on 208 activity classes are summarized in the Table 1, and the AUC values of 208 activity classes for three search approaches have been plotted in AUC heatmap in Additional file 1: Figure S4a. The AUC value of ISC is greater than the values of ISS and CSS, which is consistent with the boxplot of ∆AUCs between ISS and CSS and between ISC and CSS shown in Fig. 3. Among 208 activity classes, 178 ISS AUCs and 176 ISC AUCs are better than the corresponding CSS AUCs. Meanwhile, we also observe that there are 48 CSS AUCs smaller than 0.5, but the number is only 15 for ISS and 10 for ISC. Based on these results, we conclude that the comprehensive search performance of the algorithms is ISC > ISS > CSS.

**Fig. 4:**
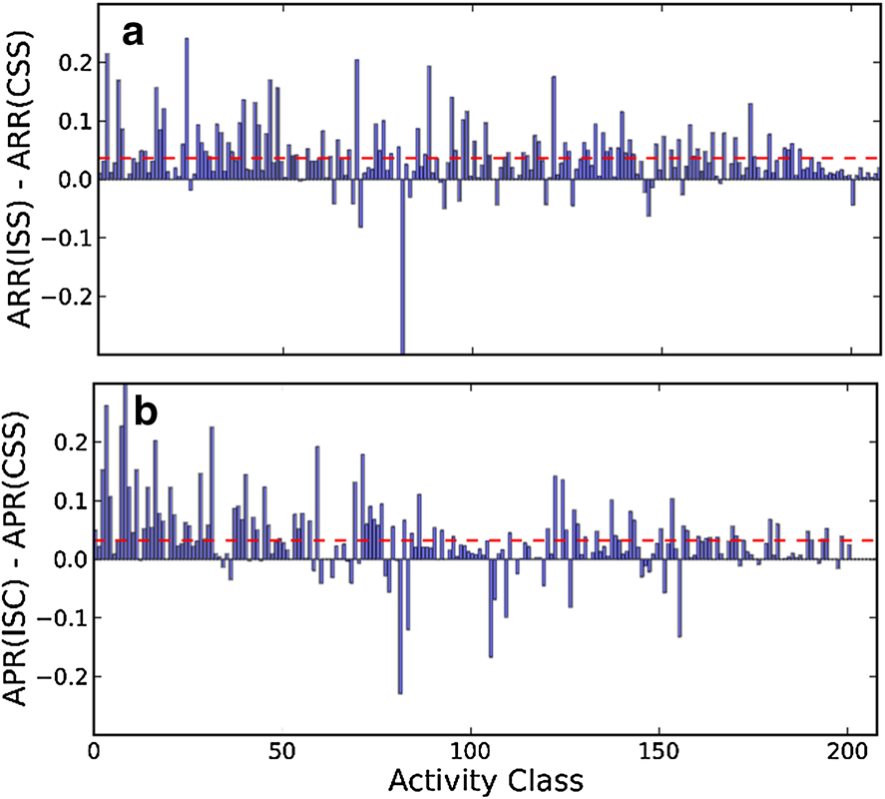
Distribution of **a** ∆ARRs of 208 activity classes between ISS and CSS, and **b** ∆APRs of 208 activity classes between ISC and CSS. The *dashed line* *in* *red color* shows the average value of 208 ∆APRs

To better understand the reason why iterative ISC and ISS search approaches outperform CSS, we compare the average recall rates (ARRs) of 208 activity classes returned by CSS, ISS and ISC. Figure 4 plots the 208 ∆ARR values between ISS and CSS, and the ∆ARRs between ISC and CSS one-by-one. ARRs of 183 activity classes returned by ISS are greater than those returned by CSS, among which ∆ARRs of 115 activity classes are statistically significant (p < 0.05) by the Mann–Whitney U test. Clearly, the ISS search approach has a much higher chance to retrieve active hits more than the CSS approach. On the other hand, ∆ARRs between ISC and CSS shows a different ∆ARR pattern in Fig. 4b, in which only 135 ISC ARRs are higher than CSS ARRs, of which 85 ISC ARRs are statistically higher than those of CSS (p < 0.05). Unlike the iterative ISS search approach, improvement of recall performance is not the major reason for the better general performance of ISC compared to ISS and CSS. This is because ISC involves inactive references in iterative search, and therefore the false-positive rejection occurs if the maximal similarity score of inactive references are higher that the maximal similarity score of active references. ~65 % of false-negative rejection happens after the similarity cutoff 0.3, which means that even when searching using the ISC search approach, a scaffold search using 2D fingerprints in a low similarity region is not suggested if the quality of search result is a priority.

**Fig. 5:**
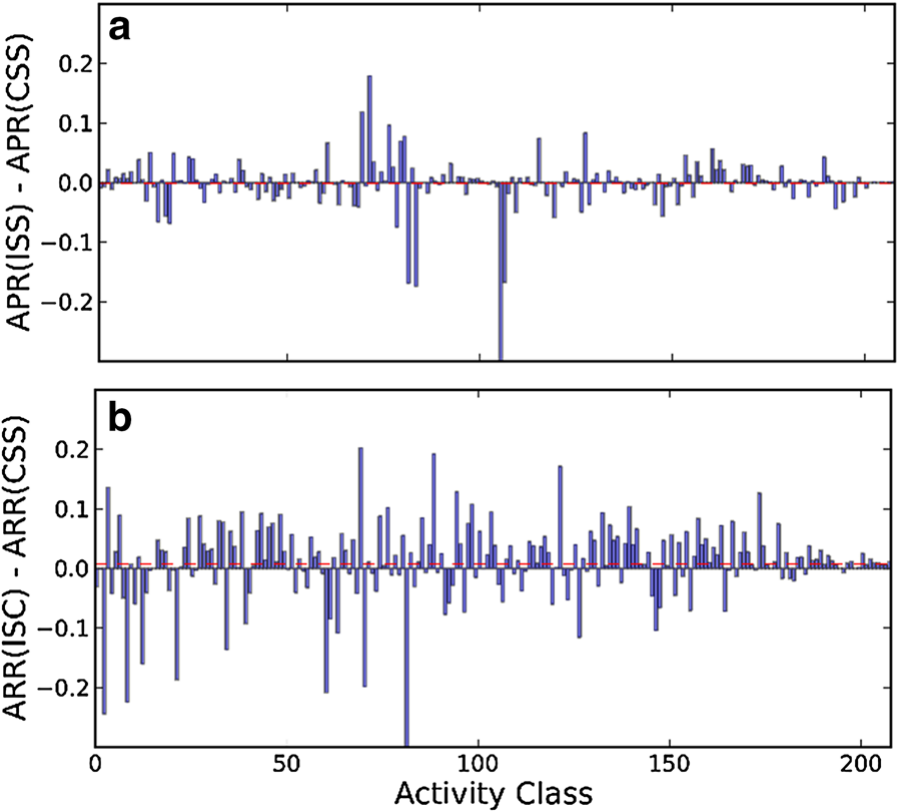
Distribution of **a** ∆APRs of 208 activity classes between ISS and CSS, and **b** ∆APRs of 208 activity classes between ISC and CSS. The *dashed line in red color* shows the average value of 208 ∆APRs

Similar comparisons are performed on ∆APRs between ISS and CSS and between ISC and CSS search approaches (Fig. 5). Although there are 120 ISS APRs higher than the corresponding CSS APRs, including 85 pairs of ∆APRs that are statistically significant by U testing, the mean value of all ∆APRs (overlapped red line) and baseline of Fig. 5a suggests that ISS and CSS generally have comparable precision performance. On the other hand, ISC shows significant better precision performance than CSS. There are 164 APRs (94 with statistically significant p < 0.05) which are higher than those of CSS. Compared to 86 activity classes on which ISS returned lower APRs than CSS, ISC failed on 44 activity classes. As a result, the mean value of 208 ∆APRs between ISC and CSS is 0.03. Clearly, significant improvement of precision is the major reason that distinguishes ISC from ISS and CSS search approaches. Furthermore, it is also interesting to observe that the ISS search approach of an iterative search with active references only improves the recall performance but not the precision performance. APRs at different similarity cutoffs (Additional file 1: Figure S3a) shows that ISS generally has slightly better precision performance than CSS in high similarity regions (i.e., Tc > 0.6 using the Morgan fingerprint) but perform worse than CSS when the search researches low similarity regions.

### Benefit of profiling in 2D similarity searches

By screening the compound structures in the bioassays, we observed that many active compounds in the same bioassay have the same scaffold. Using intermediate queries with high self-identity is one bottleneck in improving the search efficiency of iterative ISS or ISC searches. Inspired by the idea of profile searches found in sequence searches, the introduction of profiling into compound 2D similarity comparison may benefit chemical similarity searching. We chose the simple average profile (AVE) to replace the fingerprints in CSS, ISS and ISC search approaches.

**Fig. 6:**
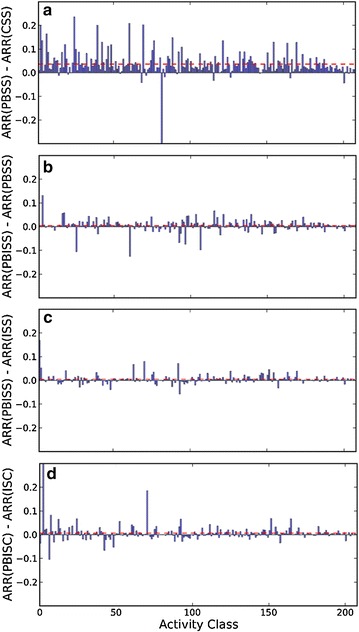
Distribution of ∆ARRs of 208 activity classes between **a** PBSS and CSS, between **b** PBISS and PBSS, between **c** PBISS and ISS, and between **d** PBISC and ISC. The *dashed line in red color* shows the average value of 208 ∆APRs

**Fig. 7:**
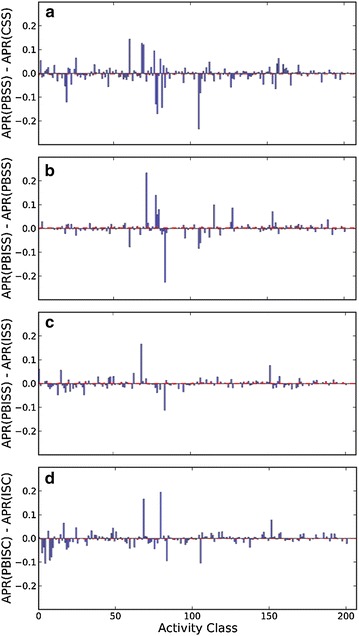
Distribution of ∆APRs of 208 activity classes between **a** PBSS and CSS, between **b** PBISS and PBSS, between **c** PBISS and ISS, and between **d** PBISC and ISC. The *dashed line in red color* shows the average value of 208 ∆APRs

AVE profile based non-iterative similarity search (PBSS) enhances the general search performance with statistical significance (p < 0.001 in Mann–Whitney U test) in comparison to CSS. 176 of 208 activity classes have PBSS AUCs greater than the corresponding AUCs of CSS search. Because an AVE profile is calculated using the fingerprints of all active references of the query compound, PBSS can also be considered as a simple bit-weighting search approach. As expected, comparisons of ∆ARRs between PBSS and CSS in Fig. 6a suggests that the recall performance of PBSS is significantly strengthened, but the precision performance between PBSS and CSS is insignificant (Fig. 7a). To improve search performance, average profiles can be implemented using vector integer instructions on modern CPUs.

**Fig. 8:**
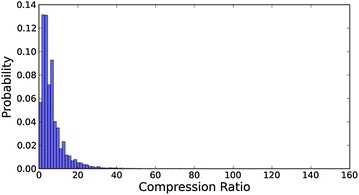
Distribution of the compression ratios of the number of references against the number of clusters for total 36,079 queries with PBISC search approach

On the other hand, introducing AVE profile into iterative ISS and ISC only slightly improves their recall performance (Fig. 6) but not their precision performance (Fig. 7), and as a result, the general search performance of PBISS and PISC does not further improve when compared to the fingerprint based ISS and ISC (Fig. 3; Table 1). The reason that profiles show limited ability to improve the recall performance in iterative searches is because fingerprints of references in the same cluster are usually of high self-identity, and therefore the newly formed profile of the cluster is still highly similar to the original fingerprint. Nevertheless, profiles do facilitate the iterative similarity search. We reviewed the clustering process of 33199 queries with the PBISC approach and we observed that the compression ratio of all queries to single profiles on average is 6.58 (Fig. 8). It should be mentioned that the maximal compression ratio reached 160, even if we limited the number of inactive compounds in the reference set and controlled the ratio of active references and inactive references to above 1:5. This suggests that profiles effectively reduce the number of comparisons in iterative search and can save computation power. Since one of the purposes of this study is to explore the potential benefits of using profiles in target enhanced 2D similarity search, the clustering and profiling procedures in our currently study were all processed on the fly. In the future, pre-clustering and pre-profiling can be performed on activity classes and the resulting profiles saved in database to facilitate profile based similarity searches.

Finally, it is worth mentioning that there is a presupposition of this study is that each query compound has at least one known binding target. However, in real world, this presupposition may be not necessary. In another word, even if the specific bioactivity of the query compound has not been confirmed, we still can use PBSS, ISS, ISC, PBISS, and PBISC search approaches to retrieve compound hits of a desired bioactivity, since the role of query compound can be regarded as the bait to fish the real compounds of desired bioactivity to form neighbor lists for further database screening. Furthermore, according to the curves of averaging 208 APRs at varied similarity cutoffs shown Additional file 1: Figure S3, PBSS can return better precision rates at high similarity cutoffs (i.e., similarity ≥0.9). This means under the extreme situation that we don’t have any knowledge of the bioactivity of the query compound, instead of using CSS to simply retrieve compounds simply based on molecular structure similarity, we can use PBSS to create the biological target profile of the query compound with high confidence, and then perform our iterative methods or use biological profile based methods like HTS-FP similarity search [33], bioturbo similarity search [34], or connectivity map [35] for more thorough virtual screening.

## Conclusion

In this paper, we introduce profiles and neighbor classification into target enhanced 2D molecular similarity searching. We have symmetrically compared the recall, precision and general search performance of two non-iterative search approaches—fingerprint based conventional similarity search (CSS) and average profile based similarity search (PBSS), two iterative search approaches with multiple active references—fingerprint based iterative search (ISS) and average profile based nearest neighbor search approaches (PBISS), two iterative search approaches with classification—fingerprint based iterative search with classification (ISC) and average profile based iterative search with classification (PBISC), a total of 6 search approaches applied to 208 activity classes.

Although the recall performance of 2D similarity search has been typically used to measure the search performance, our study suggests both recall and precision should be measured in order to evaluate search performance comprehensively. Both ISS and ISC significantly improve the recall performance but only the ISC search approach improves the precision. In addition, the introduction of profiles into 2D similarity search has two benefits. Comparing to CSS, average profiles enhance search performance. Profiles also simplify the iterative ISS and ISC search approaches without losing search performance. In balancing the recall and precision, ISC and similarly profile based ISC search approaches are promising and efficient target enhanced similarity search approaches that can be implemented in chemical databases containing bioactivity information.

## Methods

### Preparation of data sets

The PubChem Bioassay database is a large public bioactivity database, making it prudent to select data so that assay conditions should minimally bias the conclusions of this study. In our study, only bioassays containing both assay information of half-maximum inhibitory concentration (IC_50_), half-maximum effective concentration (EC_50_) or Ki values, and the explicit target sequence (GI) were systematically extracted from PubChem Bioassay database. For end-points from ChEMBL, a compound was only considered to be active when the activity concentration was below 10 μM and was only considered as inactive when the activity concentration was above 30 μM. For end-points from the PubChem confirmatory assays, the original annotations were used. Related assays were merged into an activity class if these assays had identical or similar target sequences (BLAST E value <10^−3^) and with identical screening purpose (inhibitor, antagonist, agonist et al.). In total, 2900 activity classes were created. Later an assay filtering procedure was introduced to purify to activity classes and select the final data sets for this study: (1) discard noisy activity classes if over 5 % of the target-ligand end points in the newly merged activity class were in conflict; (2) remove the conflicted pairs of end points in all of the remaining activity classes; (3) select the activity classes if both of the number of actives and the number of inactives was greater than 70. By carrying out this procedure, a database consisting of 208 activity classes including inhibitors and antagonists of designated enzymes, transporters, and receptors (Additional file 1: Table S1) was constructed. The database contained 494,199 unique compounds and 8,084,694 end points in total (Additional file 1: Table S2). A summary of 208 data sets is presented in Table 2. The large number of activity classes by itself serves to limit the effect of assay conditions on subsequent analysis.

**Table 2:** Summary of the sizes of data sets of 208 activity classes, including known actives and inactives

	Diversity index	Query set	Reference set		Test set	
			Active	Inactive	Active	Inactive
Average	6.74	144	182	200	7380	29,154
Std	1.67	202	268	278	17,984	72,132

Considering that the implementation of drug design strategies usually returns a series of compounds with high self-similarity from a single bioassay test while compounds from different bioassays have high structural diversity, we carried out the compound clustering by applying Taylor-Butina algorithm [36, 37] to cluster the active compounds in each of 208 activity classes and calculated a structure diversity index (H) by adapting Shannon’s equation (Eq. 1) [38] to represent the potential difficulty of retrieving active compounds of that activity class by given a random query compound,

1 $$H = - \mathop \sum \limits_{i = 1}^{k} \left( {\frac{{n_{i} }}{n}} \right)\log_{2} \left( {\frac{{n_{i} }}{n}} \right)$$

where k is the total number of clustering groups, n_i_ is the number of bioactive compounds in the clustering group, and n is the total number of bioactive compounds in the activity class. The larger the diversity index of that activity class, the higher the structure diversity of the active compounds in that activity class. The diversity index of 208 activity classes are listed in Additional file 1: Table S2 and their values range between 2.43 (activity classes 183) and 11.08 (activity class 147).

In order to compare the search performance of our 6 search approaches, the data set of each activity class was split into three subsets: a query set composed by annotated actives for intriguing the query procedure, a reference set for providing both active and inactive references, and a test set for evaluating the search ability of the algorithm. To ensure the structure representation of active compounds in the query set, we directly extracted the center compounds of Taylor-Butina clustering results to form the query set of every activity classes. Then we randomly assigned the remaining active compounds into the reference set and the test set. Similarly, we separated those inactive compounds in the same activity class randomly into two groups, and added them into the reference set and test set of that activity class. For the original activity classes with the number of inactive compounds exceeding 20,000, the number of inactives in the reference set was limited to one-fourth of total inactive compounds (Additional file 1: Table S2). The average sizes of query set, reference set and test set of 208 activity classes are summarized in Table 2. For each query from a selected activity class, all compounds in the query set and the reference set of the selected activity class were excluded from the database, and similarities measured between the query and all remaining compounds in the database to create the hit list for the query. All six algorithms in this study were tested with this set to ensure the validity of the comparison.

By selecting well characterized bioassay results, a large number of activity classes and compounds, ensuring structural diversity, balancing the relative weight of activity classes, and using a single test set, we attempt to ensure that our test results and conclusions are less likely affected by the varied composition of the data sets.

### Formation of average profile

Profiles have been successfully used in sequence similarity search at NCBI for many years to expand the sampling space of sequence similarity searches and to alleviate the oversampling issue in the reference set [39, 40]. In our preliminary study, we observed that some query compounds may find over 1000 neighbor compounds with both similar structure and bioactivity. It is reasonable to apply the idea of profiles in compound similarity searching by using a floating vector of the same length of the 2D fingerprint to represent the fingerprints of a group of structures or bioactivity related compounds to achieve the purpose of alleviating the search burden without losing the search sensitivity. Herein, we introduce the simple average (AVE) profile into 2D similarity search to examine whether profile based similarity searches have similar or better search performance than fingerprint based similarity searches. The general form of profile generation is.

2 $$AVE = \,\frac{{\mathop \sum \nolimits_{N} FP(i)}}{N},$$

where N represents the number of references to generate the profile, FP(i) is the fingerprint of i_th_ reference compound.

### Non-iterative similarity searches

**Fig. 9:**
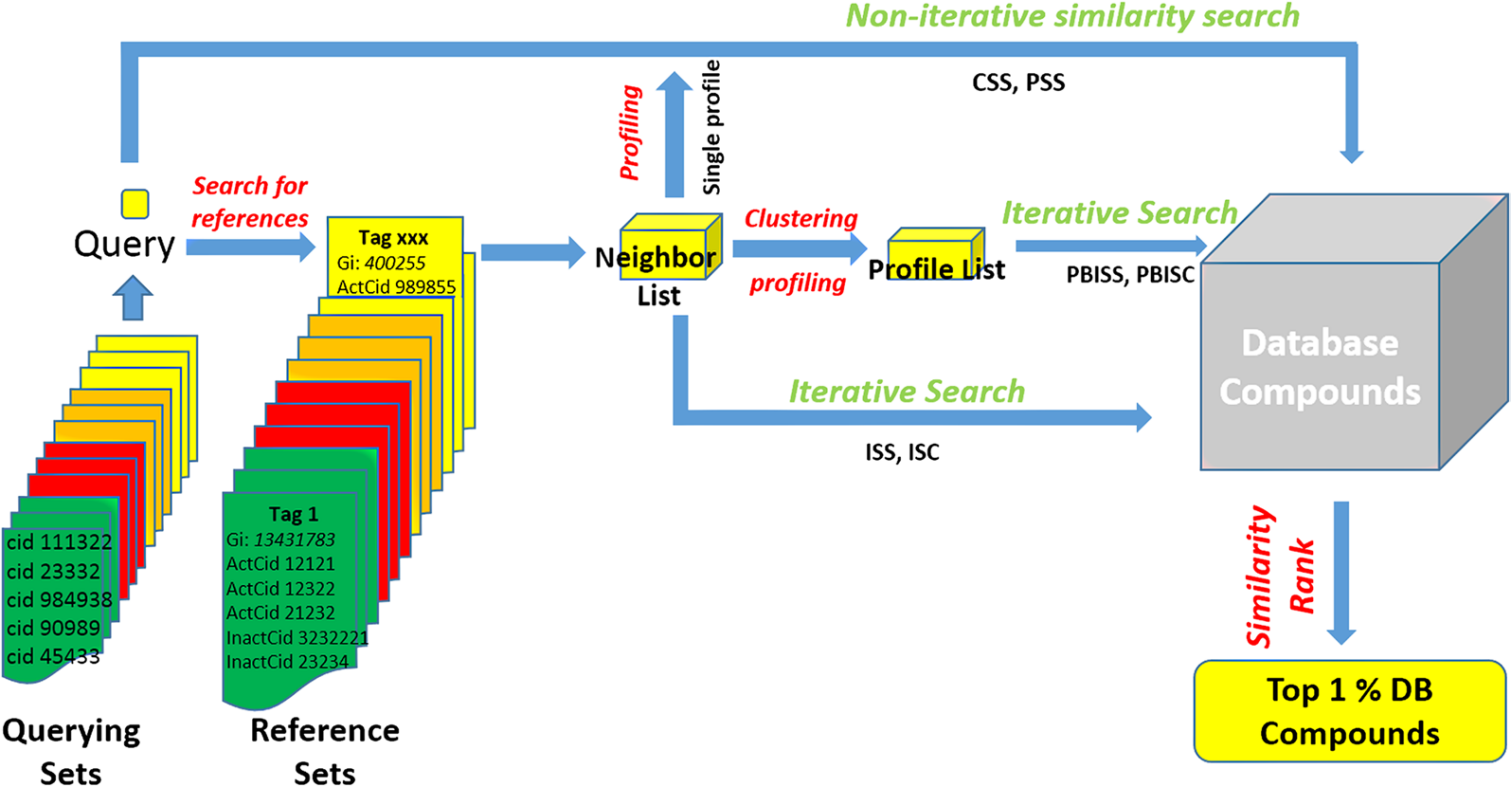
Procedures of conventional similarity search (CSS) and average profile based similarity search (PBSS), iterative nearest neighbor search (ISS) and average profile based ISS search (PBISS), and iterative search with neighbor classification (ISC) and average profile based ISC search (PBISC)

In our study, a non-iterative search is defined as only one fingerprint or one profile of the query compound participating in the similarity measurement between the query and database compound. There are two non-iterative search approaches having been systemically studied. One is 2D fingerprint based conventional similarity search (CSS) and the other is AVE profile based conventional similarity search (PBSS), which can be considered as replacing the binary fingerprint of the query compound by the AVE profile. The compound fingerprints were calculated by RDKit (Release_2013.03.2, http://redkit.org) while formation of AVE profile of the query compound required two extra steps shown by Fig. 9: (1) retrieve references with similarity score greater than 0.3 from the reference set of the assigned activity class to build a neighbor list; (2) select all of active compounds in the neighbor list to form single AVE profile by following Eq. (2). After database screening, the similarity scores of database compounds were ranked in descending order and the top 4941 hits (~1 % of database compounds) were selected as the search result of the query for further analysis.

### Iterative similarity search

Except for CSS and PBSS, fingerprint based nearest neighbor search (ISS), fingerprint based neighbor classification (ISC) and the corresponding profile versions (PBISS and PBISC) are named as iterative search approaches because at least two fingerprints/profiles participate in the similarity calculation. A brief description of the four iterative search approaches is shown in Fig. 9. Before the iterative search, all iterative search approaches first search the reference set and create the same neighbor list as the one used in the PBSS search. In iterative searches, the MAX fusion rule (max of [Tc_1_, Tc_2_, Tc_3_ …… Tc_n_ref_]) was applied in our study to assign the similarity score of database compounds. The same as in the analysis of non-iterative search results, the top 4941 hits of each query were collected for further analysis.

### ISS and ISC search approaches

**Fig. 10:**
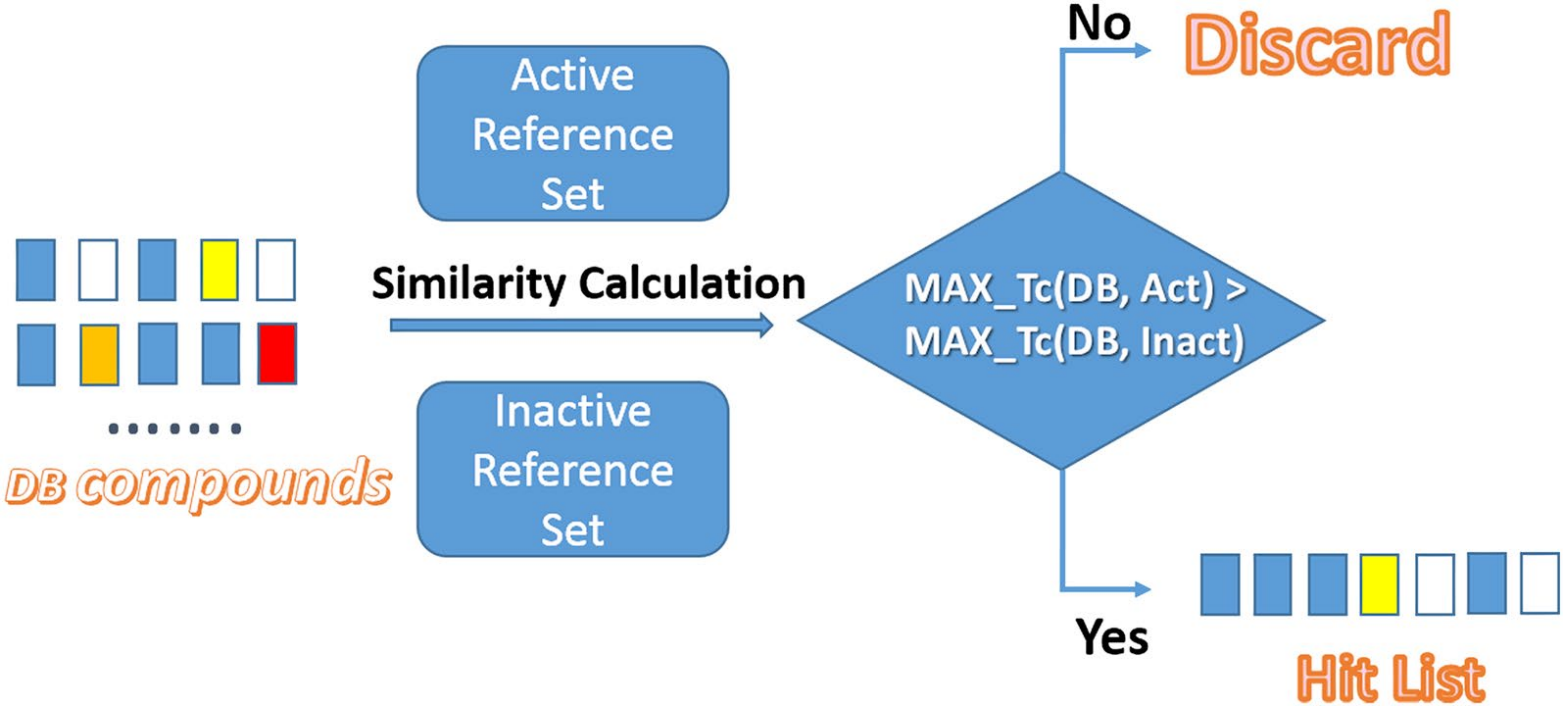
The schema of database screening of ISC and PBISC which used neighbor classification method

Instead of controlling the number of references in the iterative search as done in previous ISS searches, here we chose to control the similarity of references rather than the size of neighbor list to ensure that all structure related references are sampled. In addition, the major difference between ISS and ISC is that when querying with the ISS search approach, only active references participate in the step of iterative database screening, while ISC can be considered as the combination of ISS search with all active references and ISS search with all inactive references. As shown in Fig. 10, during the iterative database screening, if the maximal similarity between the database compound and active references was greater than the maximal similarity between the database compound and inactive references, we kept this database compound in the hit list for further analysis, otherwise we regarded this compound of high inactive potency and rejected it from the hit list. A specific example is given in Additional file 1: Figure S1 to illustrate how neighbor classification help reject the hits of high inactive potency.

### PBISS and PBISC search approaches

In our preliminary study of ISS and ISC search approaches, we observed that many reference hits to a query are of high self-similarity. Including a large amount of similar references in structure decreases the search efficiency in iterative database screening. It is for this reason we introduce the use of profiles into ISS and ISC search approaches. For the PBISS search approach, we first applied the Taylor-Butina algorithm with a similarity cutoff of 0.4 to cluster all of the active references in the neighbor list and then created one average profile for each of the clusters. For the PBISC search approach, we clustered all of references in the neighbor list of a query. If the cluster was composed of all active references or all inactive references, we created a single profile to represent the structure feature of that set of compounds. Otherwise we separated active references from inactive references and created two profiles. By using this clustering and profiling strategy, the compression ratio from fingerprints to profile is 6.58 on average from 33,199 queries.

### Fingerprint and similarity measurement

In our study, a 1024-bit hashed Morgan fingerprint, which is a circular fingerprint implemented in RDKit, was taken to characterize the structure feature of chemical compounds. The Tanimoto coefficient (Tc) [4] was chosen to measure the similarity between two fingerprints or between fingerprint and profile, as Tc similarity has been found to work well in similarity search applications [6]. The conventional form of the Tanimoto coefficient for similarity search with a binary fingerprint is defined to be

3 $$T_{c} \left( {A,B} \right) = \frac{c}{a + b - c},$$

where a and b are the number of bits set on in fingerprints of molecule A and B respectively, and c is the common bits shared by molecule A and B. The continuous form of the Tanimoto coefficient can also be applied for similarity calculation between two profiles or between a profile and a binary fingerprint. When the Tanimoto coefficient between a profile and a fingerprint is measured, the bits of the fingerprint are converted to corresponding integers “1” or “0”, and Tc can be calculated by continuous the Tanimoto Eq. 4

4 $$T_{c} \left( {A,B} \right) = \frac{{\mathop \sum \limits_{i = 1}^{M} a_{i} b_{i} }}{{\mathop \sum \limits_{i = 1}^{M} (a_{i }^{2} + b_{i}^{2} - a_{i} b_{i} )}},$$

where a_i_ and b_i_ are variables at ith position of the profiles/fingerprints of molecule A and B respectively, a_i_b_i_ is the product of a_i_ and b_i_, and M is the length of fingerprint.

### Evaluation of similarity search performance

In this study, the top 4941 hits (~top 1 % of the whole data sets) of each query on a selected activity class were analyzed, and the recall rate (RR), precision rate (PR) were also calculated. The calculation of RR follows a normal definition of recall rate (Eq. 5)

5 $$RR = \frac{{Count\,(active_{hit} )}}{{Count\,(active_{testset} )}},$$

where active_hit_ is active reference in the retrieved hit list, and active_testset_ is active reference in the test set of a selected activity class. Since the numbers of active compounds in the test sets of 208 activity classes are all smaller than 4941 compounds, the expected number of active compounds in the hit list is equal to the number of active compounds in the test set of that activity classes. On the other hand, we also count the number of inactive reference in the retrieved hit list. Therefore the calculation of PR of each query follows Eq. 6

6 $$PR = \frac{{Count\,(active_{hit} )}}{{Count\,(active_{hit} ) + Count\,(inactive_{hit} )}},$$

where inactive_hit_ is inactive reference in the retrieved hit list. The specific number of active reference and the number of inactive reference of each of 208 active classes are listed in Additional file 1: Table S2. The ARR and APR of each activity class were calculated to represent the general recall and precision performance of different search approaches on that activity class. Finally, areas under receiver operating characteristic curve (AUCs) [41] of queries on 208 activity classes were also computed.

## Additional file

10.1186/s13321-015-0103-5 The detailed information of 208 activity classes and additional figures. **Table S1** shows the target information and assay type of 208 activity classes, and **Table S2** shows the detailed compound composition of each activity classes. **Figure S3** and **Figure S4** summarize the overall APR and ARR performance of 6 similarity search approached we have studied, and **Figure S6** includes the heatmap of AUCs, the heatmap of ARRs and the heatmap of APRs of 208 activity classes returned by 6 similarity search approaches.

## Abbreviations

CSS: 2D fingerprint based conventional similarity search
ISS: 2D fingerprint based iterative similarity search
ISC: 2D fingerprint based iterative similarity search with classification
PBSS: average profile based similarity search
PBISS: average profile based iterative similarity search
PBISC: average profile based iterative similarity search with classification
AUC: area under ROC curve of the selected activity class
ARR: the arithmetic mean of recall rates of multiple queries on the selected activity class
APR: the arithmetic mean of precision rates of multiple queries on the selected activity class
