# Supplementary material for: Target enhanced 2D similarity search by using explicit biological activity annotations and profiles
Source: J Cheminform. 2015 Nov 17;7:55. doi: 10.1186/s13321-015-0103-5 (PMC4648974; doi:10.1186/s13321-015-0103-5)
Supplement: Supplementary file 1 — 10.1186/s13321-015-0103-5 The detailed information of 208 activity classes and additional figures. Table S1 shows the target information and assay type of 208 activity classes, and Table S2 shows the detailed compound composition of each activity classes. Figure S3 and Figure S4 summarize the overall APR and ARR performance of 6 similarity search approached we have studied, and Figure S6 includes the heatmap of AUCs, the heatmap of ARRs and the heatmap of APRs of 208 activity classes returned by 6 similarity search approaches. [file 13321_2015_103_MOESM1_ESM.docx]

# Table S1. Representative GI number, the type of activity class and full name of target of 208 activity classes

| **Activity Class** | **GI** | **Type** | **GI Name** |
| --- | --- | --- | --- |
| 1 | 113492 | inhibition | Type-1 angiotensin II receptor |
| 2 | 113118 | inhibition | Muscarinic acetylcholine receptor M1 |
| 3 | 128613 | inhibition | Sodium-dependent dopamine transporter; Short=DA |
| 4 | 1351851 | inhibition | Neuronal acetylcholine receptor subunit alpha-7 |
| 5 | 417418 | inhibition | Delta-type opioid receptor; Short=D-OR-1 |
| 6 | 231473 | inhibition | Adenosine receptor A1 |
| 7 | 1170947 | inhibition | Metabotropic glutamate receptor 5; Short=mGluR5 |
| 8 | 266647 | inhibition | Nitric oxide synthase |
| 9 | 125548 | inhibition | Protein kinase C alpha type |
| 10 | 400629 | inhibition | Sodium-dependent serotonin transporter |
| 11 | 548481 | inhibition | Prostaglandin G/H synthase 1 |
| 12 | 3914301 | inhibition | Prostaglandin G/H synthase 2 |
| 13 | 11141885 | inhibition | High affinity choline transporter 1 [Homo sapiens] |
| 14 | 125711 | inhibition | Proto-oncogene tyrosine-protein kinase Src |
| 15 | 120756 | inhibition | Gamma-aminobutyric acid receptor subunit alpha-2 |
| 16 | 118205 | inhibition | D(2) dopamine receptor |
| 17 | 266646 | inhibition | Nitric oxide synthase |
| 18 | 487738 | inhibition | Putative potassium channel subunit [Homo sapiens] |
| 19 | 23615263 | inhibition | m1-family aminopeptidase [Plasmodium falciparum 3D7] |
| 20 | 3183003 | inhibition | Protein-tyrosine kinase 2-beta |
| 21 | 121430 | inhibition | Glutamate receptor 1; Short=GluR-1 |
| 22 | 400254 | inhibition | Metabotropic glutamate receptor 3; Short=mGluR3 |
| 23 | 128616 | inhibition | Sodium-dependent noradrenaline transporter |
| 24 | 2498443 | inhibition | Histone deacetylase 1; Short=HD1 |
| 25 | 18027298 | inhibition | DEFINITION AKT1 [Homo sapiens] |
| 26 | 3121738 | inhibition | Steroidogenic factor 1; Short=SF-1; Short=STF-1 |
| 27 | 128997 | inhibition | Neuropeptide Y receptor type 1; Short=NPY1-R |
| 28 | 2499748 | inhibition | Dual specificity protein phosphatase 6 |
| 29 | 7582271 | inhibition | DEFINITION Mcl-1 [Homo sapiens] |
| 30 | 21264324 | inhibition | Trace amine-associated receptor 1 [Homo sapiens] |
| 31 | 1168793 | inhibition | Cathepsin K |
| 32 | 30526188 | inhibition | G-protein coupled receptor 35 [Homo sapiens] |
| 33 | 510901 | inhibition | bcl-xL [Homo sapiens] |
| 34 | 124222 | inhibition | Eukaryotic translation initiation factor 4E |
| 35 | 41872631 | inhibition | Fatty acid synthase [Homo sapiens] |
| 36 | 116670 | inhibition | Acetyl-CoA carboxylase 1; Short=ACC1 |
| 37 | 548372 | inhibition | Glutamate receptor ionotropic |
| 38 | 1352359 | inhibition | Epidermal growth factor receptor |
| 39 | 266649 | inhibition | Nitric oxide synthase |
| 40 | 11545912 | inhibition | Nucleotide-binding oligomerization domain-containing protein 2 |
| 41 | 51338793 | inhibition | Lysosomal alpha-glucosidase |
| 42 | 548377 | inhibition | Glutamate receptor ionotropic |
| 43 | 3182960 | inhibition | cGMP-specific 3' |
| 44 | 14285389 | inhibition | Potassium voltage-gated channel subfamily KQT member |
| 45 | 3041717 | inhibition | Glycogen phosphorylase |
| 46 | 21392848 | inhibition | Lethal factor [Bacillus anthracis str. A2012] |
| 47 | 20139861 | inhibition | Tyrosine-protein phosphatase non-receptor type 22 |
| 48 | 119762 | inhibition | Coagulation factor XI; Short=FXI |
| 49 | 113037 | inhibition | Acetylcholinesterase; Short=AChE |
| 50 | 23497510 | inhibition | M17 leucyl aminopeptidase [Plasmodium falciparum 3D7] |
| 51 | 4826834 | inhibition | Matrix metalloproteinase-14 preproprotein [Homo sapiens] |
| 52 | 6225311 | inhibition | Fatty-acid amide hydrolase 1 |
| 53 | 8134597 | inhibition | NADPH oxidase 1; Short=NOX-1 |
| 54 | 145559564 | inhibition | Alkaline phosphatase |
| 55 | 113979 | inhibition | Amine oxidase [flavin-containing] A |
| 56 | 68067533 | inhibition | Alkaline phosphatase |
| 57 | 113980 | inhibition | Amine oxidase [flavin-containing] B |
| 58 | 124028639 | inhibition | Amine oxidase [flavin-containing] B |
| 59 | 113105 | inhibition | Neuronal acetylcholine receptor subunit beta-2 |
| 60 | 18268203 | inhibition | Beta-secretase 1 |
| 61 | 8744934 | inhibition | X-linked inhibitor of apoptosis [Homo sapiens] |
| 62 | 31077205 | inhibition | Hepatocyte nuclear factor 4-alpha isoform HNF4alpha2 |
| 63 | 231461 | inhibition | Alpha-2A adrenergic receptor |
| 64 | 8928275 | inhibition | Photoreceptor-specific nuclear receptor |
| 65 | 1346538 | inhibition | MAP kinase-activated protein kinase 2 |
| 66 | 1352610 | inhibition | Neuropeptide Y receptor type 2; Short=NPY2-R |
| 67 | 461697 | inhibition | Cannabinoid receptor 2; Short=CB-2; Short=CB2 |
| 68 | 1346566 | inhibition | Mitogen-activated protein kinase 14; Short=MAP kinase |
| 69 | 139707 | inhibition | Protein Wnt-3a; Flags: Precursor |
| 70 | 119607128 | inhibition | Neuropeptides B/W receptor 1 [Homo sapiens] |
| 71 | 30219 | inhibition | Corticotropin releasing factor-binding protein [Homo sapiens] |
| 72 | 6912644 | inhibition | Kallikrein-5 preproprotein [Homo sapiens] |
| 73 | 135934 | inhibition | umor necrosis factor |
| 74 | 1706542 | inhibition | Dihydrofolate reductase |
| 75 | 1718191 | inhibition | Dual specificity protein phosphatase 3 |
| 76 | 22538455 | inhibition | Nuclear receptor coactivator 1 isoform 1 [Homo sapiens] |
| 77 | 32307126 | inhibition | Nuclear receptor coactivator 3 isoform a [Homo sapiens] |
| 78 | 9087218 | inhibition | Vascular endothelial growth factor receptor 2 |
| 79 | 205371758 | inhibition | Histone deacetylase 6; Short=HD6 |
| 80 | 1352513 | inhibition | Nitric oxide synthase |
| 81 | 47117888 | inhibition | cGMP-inhibited 3' |
| 82 | 6166485 | inhibition | SUMO-1-specific protease [Homo sapiens] |
| 83 | 6009644 | inhibition | Gli1 [Mus musculus] |
| 84 | 120538355 | inhibition | SUMO1/sentrin specific peptidase 7 [Homo sapiens] |
| 85 | 449081295 | inhibition | Aminopeptidase N; Short=AP-N; Short=pAPN |
| 86 | 67460301 | inhibition | Dipeptidyl peptidase 8; Short=DP8 |
| 87 | 67460390 | inhibition | Dipeptidyl peptidase 9; Short=DP9 |
| 88 | 2625023 | inhibition | Mint1 [Rattus norvegicus] |
| 89 | 45357394 | inhibition | Envelope glycoprotein [Human immunodeficiency virus 1] |
| 90 | 130316 | inhibition | Plasminogen |
| 91 | 4503131 | inhibition | Catenin beta-1 [Homo sapiens] |
| 92 | 1352311 | inhibition | Dipeptidyl peptidase 4 |
| 93 | 292495099 | inhibition | Seprase |
| 94 | 38258633 | inhibition | NAD-dependent protein deacetylase sirtuin-1 |
| 95 | 297591903 | inhibition | Large T antigen [Simian virus 40] |
| 96 | 46367787 | inhibition | Polyadenylate-binding protein 1 [Homo sapiens] |
| 97 | 14916521 | inhibition | Acetylcholinesterase; Short=AChE |
| 98 | 21361397 | inhibition | Rac GTPase-activating protein 1 [Homo sapiens] |
| 99 | 317373446 | inhibition | Coagulation factor XII |
| 100 | 116353 | inhibition | Cholinesterase |
| 101 | 75074633 | inhibition | Butyrylcholinesterase |
| 102 | 27894344 | inhibition | Nuclear receptor subfamily 4 group A member 1 isoform 1 |
| 103 | 60416391 | inhibition | Fatty-acid amide hydrolase 1 |
| 104 | 21362409 | inhibition | Cholinesterase |
| 105 | 23505220 | inhibition | M18 aspartyl aminopeptidase [Plasmodium falciparum 3D7] |
| 106 | 119576 | inhibition | Liver carboxylesterase 1 |
| 107 | 137119 | inhibition | Tissue-type plasminogen activator; Short=t-PA |
| 108 | 6686057 | inhibition | Polyphenol oxidase 2; Short=Phenolase 2 |
| 109 | 124487323 | inhibition | Intestinal alkaline phosphatase precursor [Mus musculus] |
| 110 | 124376142 | inhibition | Alkaline phosphatase |
| 111 | 113978 | inhibition | Amine oxidase [flavin-containing] A |
| 112 | 117144 | inhibition | Cytochrome P450 1A2; AltName: Full=CYPIA2 |
| 113 | 117293 | inhibition | Aromatase; AltName: Full=CYPXIX |
| 114 | 116241312 | inhibition | Cytochrome P450 3A4 |
| 115 | 84028191 | inhibition | Cytochrome P450 2D6; AltName: Full=CYPIID6 |
| 116 | 4504343 | inhibition | Nuclear receptor subfamily 5 group A member 2 isoform 2 |
| 117 | 15608158 | inhibition | Probable UDP-N-acetylglucosamine pyrophosphorylase GlmU |
| 118 | 136620 | inhibition | Trypanothione reductase |
| 119 | 212276510 | inhibition | Dipeptidyl peptidase 2 |
| 120 | 9629429 | inhibition | Transactivating tegument protein VP16 [Human herpesvirus 1] |
| 121 | 119761 | inhibition | Coagulation factor X |
| 122 | 2493531 | inhibition | Caspase-8; Short=CASP-8 |
| 123 | 123271505 | inhibition | Heat shock 70kDa protein 1A [Homo sapiens] |
| 124 | 70832125 | inhibition | Hexokinase [Trypanosoma brucei brucei strain 927/4 GUTat10.1]. |
| 125 | 116852 | inhibition | Interstitial collagenase |
| 126 | 2507196 | inhibition | Mitogen-activated protein kinase 10; Short=MAP kinase |
| 127 | 83300925 | inhibition | Carbonic anhydrase 9 |
| 128 | 131467 | inhibition | Tyrosine-protein phosphatase non-receptor type 1 |
| 129 | 543740 | inhibition | Adenosine receptor A2a |
| 130 | 30316367 | inhibition | Corticosteroid 11-beta-dehydrogenase isozyme 2 |
| 131 | 83287886 | inhibition | Prostaglandin E2 receptor EP1 subtype; Short=PGE |
| 132 | 18375660 | inhibition | Tyrosine-protein phosphatase non-receptor type 7 isoform 2 |
| 133 | 56405372 | inhibition | Dihydroorotate dehydrogenase (quinone) |
| 134 | 115298672 | inhibition | Neutrophil cytosol factor 1 [Homo sapiens] |
| 135 | 38258608 | inhibition | NAD-dependent protein deacetylase sirtuin-2 |
| 136 | 13399304 | inhibition | DNA dC->dU-editing enzyme APOBEC-3G [Homo sapiens] |
| 137 | 16878311 | inhibition | MPI protein [Homo sapiens] |
| 138 | 112938 | inhibition | Adenosine receptor A2b |
| 139 | 399777 | inhibition | Gonadotropin-releasing hormone receptor; Short=GnRH |
| 140 | 33695107 | inhibition | G-protein coupled receptor 55 [Homo sapiens] |
| 141 | 113984 | inhibition | DNA-(apurinic or apyrimidinic site) lyase |
| 142 | 1351831 | inhibition | Adenosine receptor A3 |
| 143 | 1168407 | inhibition | Aldose reductase; Short=AR |
| 144 | 115465 | inhibition | Carbonic anhydrase 4 |
| 145 | 461680 | inhibition | Carbonic anhydrase 5A |
| 146 | 116051 | inhibition | Cyclin-dependent kinase 2 |
| 147 | 219689097 | inhibition | Phosphoethanolamine/phosphocholine phosphatase isoform 1 |
| 148 | 219689243 | inhibition | Chain A |
| 149 | 119292 | inhibition | Bone marrow serine |
| 150 | 115449 | inhibition | Carbonic anhydrase 1 |
| 151 | 47678753 | inhibition | XBP1 [Homo sapiens] |
| 152 | 115456 | inhibition | Carbonic anhydrase 2 |
| 153 | 347595814 | inhibition | Deoxyuridine 5'-triphosphate nucleotidohydrolase |
| 154 | 81952007 | inhibition | NS5B protein |
| 155 | 45219878 | inhibition | Eukaryotic translation initiation factor 4H [Homo sapiens] |
| 156 | 139424501 | inhibition | BZLF2 [Human herpesvirus 4 type 2] |
| 157 | 227977143 | inhibition | nonstructural protein 1 |
| 158 | 262118306 | inhibition | Sentrin-specific protease 8 [Homo sapiens] |
| 159 | 21955158 | inhibition | DNA dC->dU-editing enzyme APOBEC-3A isoform a [Homo sapiens] |
| 160 | 118992 | inhibition | Dihydrofolate reductase |
| 161 | 11024684 | inhibition | Bcl-2-related protein A1 [Mus musculus] |
| 162 | 160707929 | inhibition | DNA damage-inducible transcript 3 protein [Mus musculus] |
| 163 | 4507785 | inhibition | SUMO-conjugating enzyme UBC9 [Homo sapiens] |
| 164 | 4507793 | inhibition | Ubiquitin-conjugating enzyme E2 N [Homo sapiens] |
| 165 | 75593047 | inhibition | Pol polyprotein |
| 166 | 285809906 | inhibition | TPA: Tim10p |
| 167 | 13272532 | inhibition | STAT3 |
| 168 | 464314 | activation | Mu-type opioid receptor; Short=M-OR-1; Short=MOR-1 |
| 169 | 115562 | activation | Cannabinoid receptor 1; Short=CB-R; Short=CB1 |
| 170 | 125374 | activation | Glycogen synthase kinase-3 beta; Short=GSK-3 beta |
| 171 | 464312 | activation | Kappa-type opioid receptor; Short=K-OR-1 |
| 172 | 2494982 | activation | Melanocortin receptor 4; Short=MC4-R |
| 173 | 14670397 | activation | T-type calcium channel alpha 1H subunit [Homo sapiens] |
| 174 | 487738 | activation | Putative potassium channel subunit [Homo sapiens] |
| 175 | 547736 | activation | ATP-sensitive inward rectifier potassium channel 1 |
| 176 | 21264324 | activation | Trace amine-associated receptor 1 [Homo sapiens] |
| 177 | 42741659 | activation | Multidrug resistance protein 1 [Homo sapiens] |
| 178 | 12003227 | activation | Electroneutral potassium-chloride cotransporter KCC2 |
| 179 | 11545912 | activation | Nucleotide-binding oligomerization domain-containing protein 2 |
| 180 | 21542418 | activation | Nuclear factor NF-kappa-B p105 subunit |
| 181 | 5174617 | activation | Nucleotide-binding oligomerization domain-containing protein 1 |
| 182 | 14285389 | activation | Potassium voltage-gated channel subfamily KQT member |
| 183 | 17380296 | activation | Sodium- and chloride-dependent glycine transporter 2 |
| 184 | 1174458 | activation | Signal transducer and activator of transcription 1 |
| 185 | 145559564 | activation | Alkaline phosphatase |
| 186 | 68067533 | activation | Alkaline phosphatase |
| 187 | 17541622 | activation | Protein MEX-5 [Caenorhabditis elegans] |
| 188 | 15929025 | activation | Sphingosine-1-phosphate receptor 4 [Homo sapiens]. |
| 189 | 38788193 | activation | Sphingosine 1-phosphate receptor 3 [Homo sapiens]. |
| 190 | 461697 | activation | Cannabinoid receptor 2; Short=CB-2; Short=CB2; |
| 191 | 30219 | activation | Corticotropin releasing factor-binding protein [Homo sapiens]. |
| 192 | 2493280 | activation | Bcl-2-related protein A1; AltName: Full=Bcl-2-like |
| 193 | 139472804 | activation | LANA [Human herpesvirus 8]. |
| 194 | 160333370 | activation | Short transient receptor potential channel 6 isoform 1 |
| 195 | 297591903 | activation | Large T antigen [Simian virus 40]. |
| 196 | 124487323 | activation | Intestinal alkaline phosphatase precursor [Mus musculus]. |
| 197 | 119609447 | activation | Karyopherin alpha 2 |
| 198 | 124376142 | activation | Alkaline phosphatase |
| 199 | 486173 | activation | LAP4 [Saccharomyces cerevisiae]. |
| 200 | 62740231 | activation | Hsf1 protein [Mus musculus]. |
| 201 | 38174238 | activation | MCOLN3 protein [Homo sapiens]. |
| 202 | 33695107 | activation | G-protein coupled receptor 55 [Homo sapiens]. |
| 203 | 1927 | activation | Cardiac alpha tropomyosin [Sus scrofa]. |
| 204 | 17562800 | activation | Protein POS-1 [Caenorhabditis elegans]. |
| 205 | 20336315 | activation | Bcl-2-like protein 11 isoform 1 [Homo sapiens]. |
| 206 | 31933 | activation | Glutathione S-transferase |
| 207 | 536029 | activation | RPL19A [Saccharomyces cerevisiae]. |
| 208 | 6690534 | activation | Matrix metalloproteinase 1 |

# Table S2. The query size, diversity index, and active and inactive compound numbers of each of 208 activity classes in database

| Activity Class | Diversity Index | Query Set | Reference Set | | Test Set | |
| --- | --- | --- | --- | --- | --- | --- |
|  |  |  | Act_Ref | Inact_Ref | Act_Test | Inact_Test |
| 1 | 2.43 | 5 | 35 | 39 | 64 | 37 |
| 2 | 2.65 | 9 | 31 | 87 | 94 | 113 |
| 3 | 3.15 | 5 | 20 | 32 | 50 | 22 |
| 4 | 3.70 | 16 | 33 | 39 | 77 | 44 |
| 5 | 3.73 | 17 | 30 | 39 | 37 | 43 |
| 6 | 3.73 | 16 | 47 | 63 | 51 | 26 |
| 7 | 3.75 | 31 | 61 | 115 | 84 | 82 |
| 8 | 3.77 | 15 | 19 | 42 | 47 | 40 |
| 9 | 3.90 | 21 | 26 | 34 | 65 | 60 |
| 10 | 3.92 | 15 | 23 | 36 | 39 | 32 |
| 11 | 3.99 | 20 | 36 | 42 | 43 | 39 |
| 12 | 4.05 | 25 | 46 | 67 | 35 | 46 |
| 13 | 4.05 | 18 | 26 | 43 | 39011 | 156615 |
| 14 | 4.05 | 26 | 27 | 39 | 59 | 41 |
| 15 | 4.28 | 10 | 24 | 27 | 72 | 53 |
| 16 | 4.28 | 32 | 90 | 114 | 37 | 34 |
| 17 | 4.41 | 29 | 51 | 63 | 176 | 202 |
| 18 | 4.61 | 43 | 97 | 124 | 64 | 63 |
| 19 | 4.65 | 21 | 20 | 22 | 48 | 49 |
| 20 | 4.69 | 20 | 11 | 23 | 65 | 57 |
| 21 | 4.70 | 16 | 41 | 51 | 52 | 55 |
| 22 | 4.71 | 25 | 83 | 106 | 81 | 99 |
| 23 | 4.82 | 40 | 118 | 157 | 72 | 70 |
| 24 | 4.82 | 55 | 97 | 146 | 51 | 50 |
| 25 | 4.84 | 22 | 25 | 42 | 113 | 104 |
| 26 | 4.84 | 9 | 11 | 18 | 60 | 71 |
| 27 | 4.87 | 26 | 41 | 49 | 42 | 59 |
| 28 | 4.88 | 55 | 105 | 154 | 43 | 46 |
| 29 | 4.90 | 24 | 52 | 67 | 58 | 72 |
| 30 | 4.92 | 49 | 290 | 329 | 21 | 62 |
| 31 | 4.95 | 40 | 87 | 108 | 40 | 35 |
| 32 | 4.96 | 41 | 72 | 91 | 98 | 104 |
| 33 | 4.98 | 48 | 126 | 153 | 44 | 52 |
| 34 | 5.09 | 63 | 346 | 392 | 112 | 140 |
| 35 | 5.10 | 24 | 39 | 44 | 12984 | 51882 |
| 36 | 5.12 | 51 | 222 | 274 | 45 | 45 |
| 37 | 5.14 | 47 | 94 | 127 | 50 | 62 |
| 38 | 5.15 | 13 | 23 | 45 | 54 | 47 |
| 39 | 5.20 | 35 | 40 | 57 | 59 | 89 |
| 40 | 5.21 | 58 | 77 | 88 | 41 | 41 |
| 41 | 5.24 | 45 | 71 | 83 | 166 | 148 |
| 42 | 5.31 | 72 | 179 | 279 | 56901 | 227191 |
| 43 | 5.47 | 17 | 19 | 25 | 108 | 109 |
| 44 | 5.49 | 83 | 205 | 234 | 71 | 67 |
| 45 | 5.49 | 70 | 201 | 217 | 40 | 39 |
| 46 | 5.49 | 53 | 86 | 99 | 78 | 68 |
| 47 | 5.49 | 61 | 97 | 107 | 155 | 125 |
| 48 | 5.50 | 44 | 51 | 73 | 46 | 39 |
| 49 | 5.53 | 57 | 82 | 95 | 51 | 60 |
| 50 | 5.54 | 77 | 200 | 276 | 60 | 67 |
| 51 | 5.56 | 24 | 39 | 48 | 154 | 153 |
| 52 | 5.59 | 50 | 110 | 125 | 42 | 46 |
| 53 | 5.60 | 18 | 10 | 11 | 58363 | 233983 |
| 54 | 5.61 | 36 | 63 | 76 | 83 | 75 |
| 55 | 5.62 | 26 | 94 | 125 | 55 | 66 |
| 56 | 5.62 | 54 | 70 | 100 | 48 | 93 |
| 57 | 5.64 | 26 | 33 | 56 | 49 | 54 |
| 58 | 5.64 | 78 | 141 | 147 | 53 | 56 |
| 59 | 5.66 | 64 | 132 | 150 | 33 | 45 |
| 60 | 5.71 | 84 | 110 | 132 | 183 | 163 |
| 61 | 5.72 | 3 | 6 | 8 | 200 | 214 |
| 62 | 5.74 | 25 | 11 | 17 | 26470 | 106119 |
| 63 | 5.83 | 27 | 20 | 39 | 130 | 138 |
| 64 | 5.89 | 10 | 10 | 12 | 362 | 385 |
| 65 | 5.90 | 93 | 248 | 289 | 29 | 42 |
| 66 | 5.91 | 85 | 149 | 171 | 233 | 255 |
| 67 | 5.92 | 101 | 105 | 155 | 44 | 55 |
| 68 | 5.94 | 92 | 121 | 139 | 33 | 38 |
| 69 | 5.96 | 4 | 5 | 6 | 241 | 245 |
| 70 | 6.00 | 25 | 13 | 17 | 591 | 560 |
| 71 | 6.02 | 25 | 12 | 25 | 19371 | 76961 |
| 72 | 6.03 | 4 | 14 | 23 | 171 | 154 |
| 73 | 6.04 | 18 | 18 | 27 | 57 | 99 |
| 74 | 6.04 | 63 | 91 | 108 | 132 | 144 |
| 75 | 6.05 | 147 | 268 | 343 | 140 | 120 |
| 76 | 6.07 | 62 | 31 | 35 | 74 | 56 |
| 77 | 6.12 | 48 | 36 | 46 | 330 | 282 |
| 78 | 6.14 | 11 | 13 | 17 | 171 | 141 |
| 79 | 6.19 | 5 | 9 | 9 | 291 | 309 |
| 80 | 6.20 | 6 | 12 | 16 | 74 | 82 |
| 81 | 6.20 | 6 | 8 | 21 | 80 | 88 |
| 82 | 6.20 | 2 | 3 | 4 | 663 | 664 |
| 83 | 6.21 | 97 | 171 | 200 | 99 | 91 |
| 84 | 6.25 | 14 | 17 | 28 | 45 | 73 |
| 85 | 6.28 | 75 | 124 | 145 | 105 | 99 |
| 86 | 6.30 | 175 | 498 | 591 | 37 | 39 |
| 87 | 6.31 | 52 | 49 | 52 | 185 | 175 |
| 88 | 6.34 | 136 | 355 | 373 | 44 | 43 |
| 89 | 6.37 | 185 | 532 | 588 | 166 | 161 |
| 90 | 6.41 | 106 | 175 | 182 | 69 | 82 |
| 91 | 6.42 | 110 | 242 | 267 | 93 | 93 |
| 92 | 6.45 | 16 | 22 | 26 | 58385 | 233146 |
| 93 | 6.46 | 57 | 33 | 39 | 45 | 29 |
| 94 | 6.46 | 7 | 5 | 5 | 19222 | 77084 |
| 95 | 6.46 | 134 | 197 | 224 | 35 | 46 |
| 96 | 6.52 | 137 | 294 | 310 | 188 | 187 |
| 97 | 6.52 | 16 | 9 | 17 | 64 | 74 |
| 98 | 6.57 | 238 | 1055 | 1098 | 45 | 101 |
| 99 | 6.59 | 192 | 476 | 500 | 51 | 53 |
| 100 | 6.61 | 61 | 99 | 127 | 587 | 560 |
| 101 | 6.65 | 177 | 592 | 622 | 53 | 55 |
| 102 | 6.66 | 59 | 33 | 43 | 57 | 33 |
| 103 | 6.67 | 174 | 309 | 343 | 41 | 30 |
| 104 | 6.68 | 221 | 822 | 945 | 45 | 50 |
| 105 | 6.69 | 138 | 394 | 458 | 108 | 115 |
| 106 | 6.70 | 2 | 29 | 37 | 64 | 63 |
| 107 | 6.70 | 57 | 23 | 26 | 123 | 102 |
| 108 | 6.72 | 137 | 210 | 227 | 83 | 75 |
| 109 | 6.73 | 179 | 376 | 404 | 89 | 89 |
| 110 | 6.74 | 34 | 14 | 23 | 55 | 81 |
| 111 | 6.74 | 57 | 74 | 81 | 110 | 97 |
| 112 | 6.74 | 24 | 19 | 32 | 58390 | 233378 |
| 113 | 6.76 | 14 | 32 | 38 | 444 | 427 |
| 114 | 6.85 | 172 | 209 | 263 | 58 | 53 |
| 115 | 6.91 | 162 | 237 | 259 | 96 | 105 |
| 116 | 6.92 | 11 | 10 | 11 | 445 | 460 |
| 117 | 6.94 | 46 | 30 | 32 | 39067 | 156615 |
| 118 | 6.98 | 43 | 15 | 17 | 167 | 165 |
| 119 | 6.99 | 95 | 75 | 85 | 13013 | 51859 |
| 120 | 7.00 | 9 | 10 | 13 | 260 | 279 |
| 121 | 7.04 | 100 | 202 | 241 | 187 | 172 |
| 122 | 7.09 | 228 | 525 | 588 | 106 | 103 |
| 123 | 7.10 | 55 | 43 | 62 | 88 | 100 |
| 124 | 7.15 | 41 | 26 | 31 | 22479 | 89912 |
| 125 | 7.17 | 43 | 27 | 30 | 96 | 83 |
| 126 | 7.19 | 231 | 462 | 478 | 237 | 254 |
| 127 | 7.19 | 13 | 11 | 12 | 840 | 891 |
| 128 | 7.22 | 23 | 40 | 42 | 80 | 85 |
| 129 | 7.25 | 46 | 23 | 31 | 108 | 102 |
| 130 | 7.25 | 255 | 593 | 627 | 32 | 43 |
| 131 | 7.25 | 242 | 369 | 393 | 108 | 108 |
| 132 | 7.26 | 53 | 39 | 47 | 38733 | 155267 |
| 133 | 7.28 | 251 | 387 | 406 | 91 | 89 |
| 134 | 7.32 | 133 | 293 | 331 | 199 | 192 |
| 135 | 7.35 | 326 | 994 | 1051 | 180 | 202 |
| 136 | 7.35 | 257 | 339 | 399 | 76 | 67 |
| 137 | 7.37 | 306 | 814 | 833 | 62 | 69 |
| 138 | 7.40 | 15 | 15 | 17 | 413 | 436 |
| 139 | 7.40 | 332 | 813 | 808 | 130 | 138 |
| 140 | 7.40 | 316 | 530 | 548 | 85 | 94 |
| 141 | 7.47 | 252 | 317 | 333 | 72 | 63 |
| 142 | 7.49 | 345 | 902 | 944 | 105 | 106 |
| 143 | 7.49 | 204 | 252 | 261 | 317 | 374 |
| 144 | 7.50 | 172 | 172 | 203 | 40 | 39 |
| 145 | 7.51 | 203 | 306 | 313 | 39 | 44 |
| 146 | 7.53 | 16 | 18 | 23 | 71 | 91 |
| 147 | 7.53 | 8 | 11 | 12 | 723 | 790 |
| 148 | 7.54 | 86 | 30 | 45 | 77 | 70 |
| 149 | 7.55 | 338 | 866 | 928 | 84 | 49 |
| 150 | 7.57 | 134 | 152 | 168 | 809 | 796 |
| 151 | 7.57 | 263 | 425 | 439 | 196 | 191 |
| 152 | 7.63 | 8 | 10 | 11 | 87 | 82 |
| 153 | 7.74 | 395 | 989 | 1056 | 137 | 94 |
| 154 | 7.77 | 31 | 18 | 31 | 213 | 194 |
| 155 | 7.79 | 306 | 342 | 364 | 56 | 48 |
| 156 | 7.87 | 12 | 11 | 13 | 388 | 427 |
| 157 | 7.87 | 30 | 23 | 31 | 38 | 48 |
| 158 | 7.92 | 64 | 32 | 36 | 257 | 298 |
| 159 | 7.97 | 383 | 810 | 809 | 33 | 39 |
| 160 | 7.99 | 510 | 1229 | 1308 | 76 | 88 |
| 161 | 7.99 | 29 | 21 | 24 | 71 | 75 |
| 162 | 8.03 | 174 | 63 | 76 | 39 | 42 |
| 163 | 8.04 | 99 | 48 | 53 | 285 | 321 |
| 164 | 8.09 | 374 | 424 | 445 | 162 | 124 |
| 165 | 8.10 | 49 | 27 | 41 | 57643 | 230961 |
| 166 | 8.11 | 15 | 18 | 21 | 304 | 302 |
| 167 | 8.12 | 457 | 898 | 984 | 59 | 56 |
| 168 | 8.13 | 40 | 37 | 39 | 58167 | 232916 |
| 169 | 8.20 | 7 | 20 | 21 | 293 | 294 |
| 170 | 8.26 | 429 | 442 | 471 | 180 | 148 |
| 171 | 8.30 | 73 | 35 | 44 | 198 | 181 |
| 172 | 8.35 | 96 | 51 | 59 | 932 | 1006 |
| 173 | 8.39 | 100 | 52 | 61 | 135 | 112 |
| 174 | 8.42 | 642 | 1119 | 1152 | 47 | 67 |
| 175 | 8.45 | 661 | 1562 | 1560 | 62 | 55 |
| 176 | 8.49 | 133 | 59 | 64 | 17275 | 68922 |
| 177 | 8.53 | 95 | 67 | 69 | 156 | 138 |
| 178 | 8.54 | 158 | 73 | 77 | 38491 | 154414 |
| 179 | 8.74 | 345 | 179 | 207 | 43 | 33 |
| 180 | 8.78 | 66 | 55 | 67 | 426 | 418 |
| 181 | 8.78 | 316 | 109 | 123 | 97 | 112 |
| 182 | 8.81 | 123 | 78 | 85 | 309 | 332 |
| 183 | 8.83 | 68 | 25 | 27 | 43021 | 172261 |
| 184 | 8.87 | 286 | 158 | 160 | 58189 | 233253 |
| 185 | 8.92 | 284 | 123 | 136 | 22358 | 89528 |
| 186 | 8.92 | 196 | 64 | 69 | 142 | 163 |
| 187 | 8.99 | 329 | 136 | 144 | 43155 | 172993 |
| 188 | 9.01 | 226 | 112 | 122 | 58 | 57 |
| 189 | 9.02 | 245 | 118 | 139 | 58084 | 231819 |
| 190 | 9.03 | 356 | 116 | 119 | 214 | 186 |
| 191 | 9.04 | 169 | 88 | 89 | 405 | 432 |
| 192 | 9.06 | 318 | 161 | 173 | 67120 | 268950 |
| 193 | 9.07 | 267 | 83 | 90 | 94 | 95 |
| 194 | 9.07 | 781 | 1469 | 1466 | 600 | 553 |
| 195 | 9.12 | 209 | 74 | 78 | 240 | 233 |
| 196 | 9.14 | 309 | 156 | 160 | 66905 | 267212 |
| 197 | 9.27 | 273 | 120 | 132 | 42984 | 172293 |
| 198 | 9.64 | 202 | 57 | 61 | 69 | 55 |
| 199 | 9.66 | 386 | 146 | 152 | 550 | 544 |
| 200 | 9.73 | 427 | 118 | 124 | 60456 | 242134 |
| 201 | 9.97 | 72 | 12 | 12 | 1553 | 1646 |
| 202 | 10.06 | 684 | 283 | 287 | 58046 | 232450 |
| 203 | 10.32 | 952 | 260 | 275 | 67541 | 270570 |
| 204 | 10.48 | 332 | 61 | 69 | 58118 | 231534 |
| 205 | 10.93 | 945 | 249 | 261 | 57019 | 228529 |
| 206 | 10.94 | 1202 | 353 | 366 | 57004 | 229430 |
| 207 | 11.03 | 964 | 266 | 277 | 57161 | 228810 |
| 208 | 11.08 | 1282 | 337 | 351 | 57179 | 228024 |

Figure S1.


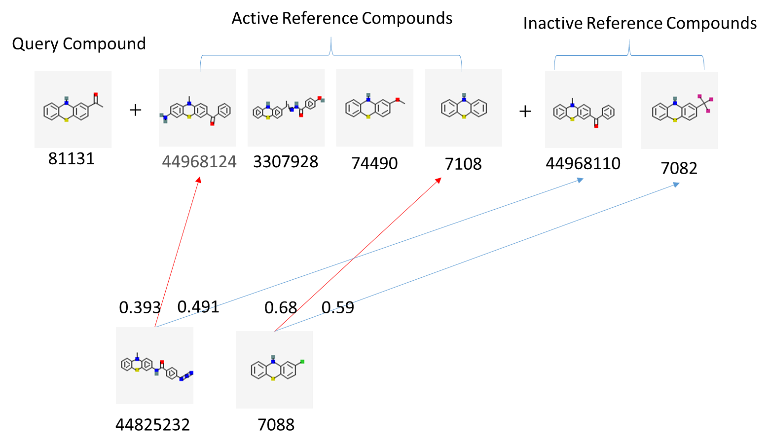


Accept

Reject

# Figure S2. ARRs against the similarity cutoffs

**(b)**


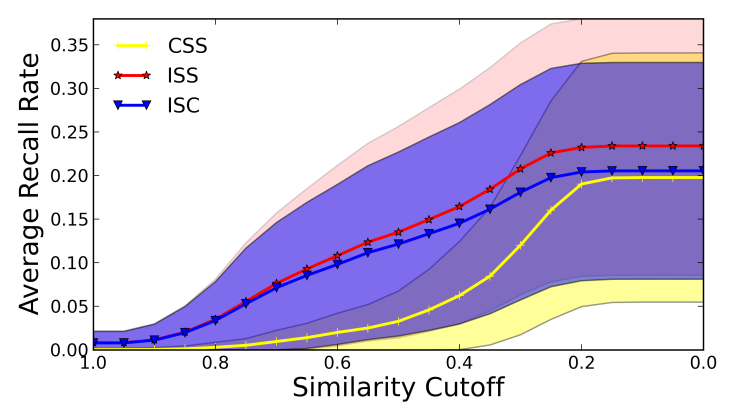


**(a)**


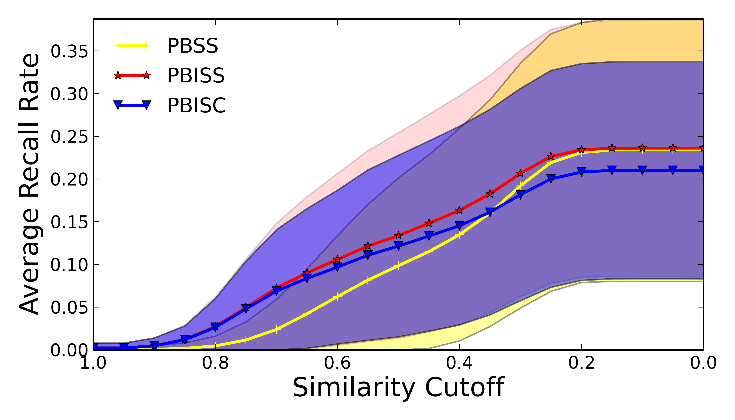


# Figure S3. APRs against the similarity cutoffs


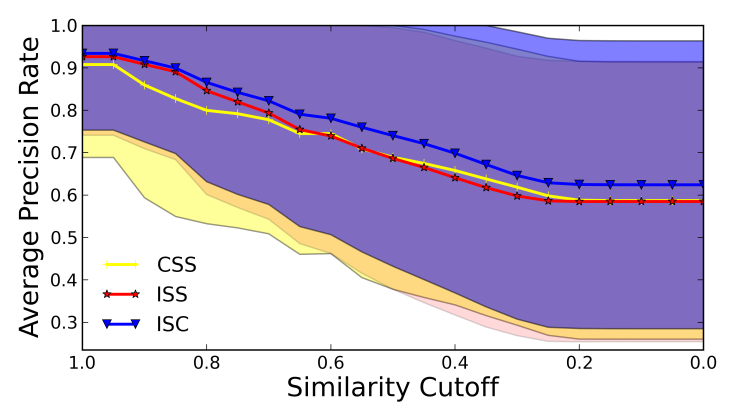


**(b)**

**(a)**


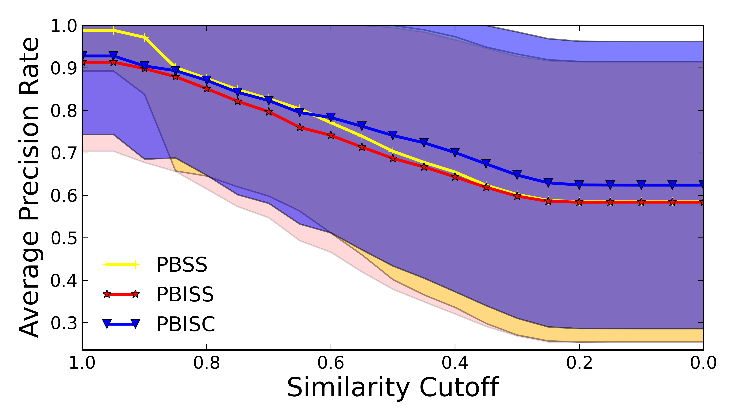


# Figure S4. (a) The heatmap of AUCs, (b) the heatmap of ARRs, and (c) the heatmap of APRs of 208 activity classes. The columns from 1 to 6 represent for the 208 values returned by 1-CSS, 2-PBSS, 3-ISS, 4-PBISS, 5-ISC, and 6-PBISC.


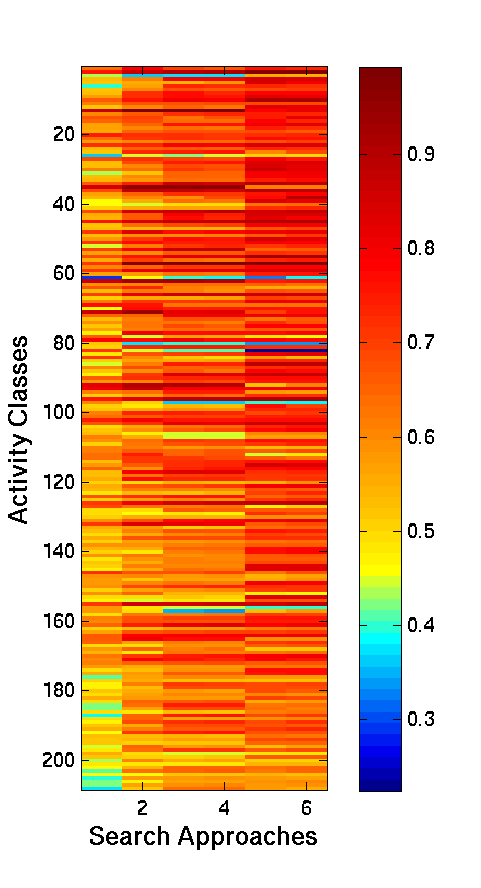

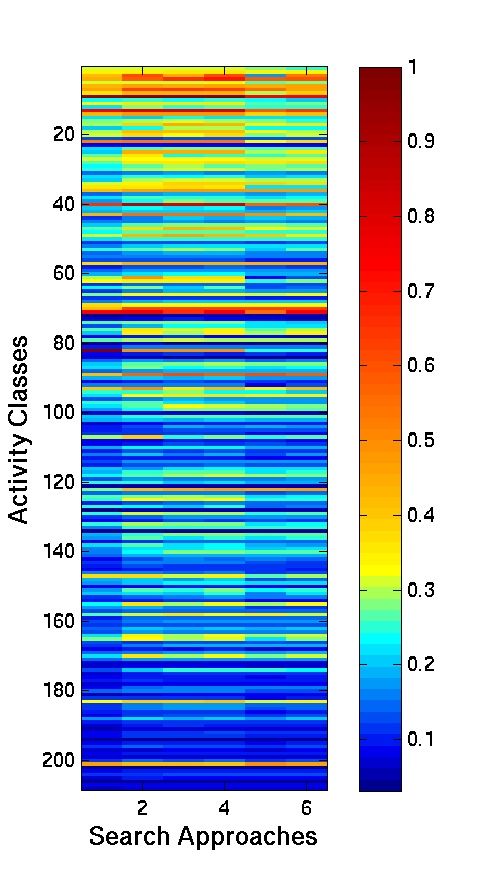

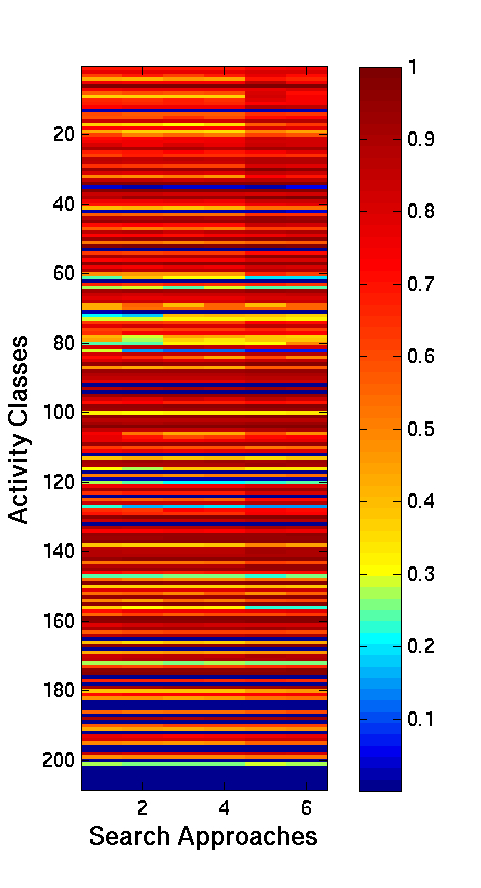


**(c)**

**(a)**

**(b)**
